# 1-Methyl-3-(naphthalen-2-yl)cyclo­penta­diene

**DOI:** 10.1107/S2414314623008568

**Published:** 2023-10-10

**Authors:** Melina Michailidis, Peter J. Bonitatibus Jr

**Affiliations:** a Rensselaer Polytechnic Institute, Department of Chemistry and Chemical Biology, Cogswell Laboratory, 110 8th Street, Troy, NY 12180, USA; Purdue University, USA

**Keywords:** crystal structure, cyclo­penta­diene, luminescence sensitization

## Abstract

The title compound, C_16_H_14_, an asymmetric naphthyl-/methyl-substituted cyclo­penta­diene was synthesized and one isomer of five accessible through sigmatropic rearrangement was isolated and characterized by ^1^H NMR and X-ray diffraction. The crystal packing features an inter­molecular C—H⋯π inter­action.

## Abstract 2

An asymmetric naphthyl-/methyl-substituted cyclo­penta­diene was synthesized and one isomer of five accessible through sigmatropic rearrangement was isolated and characterized by ^1^H NMR and X-ray diffraction. The title compound is envisioned as a π-bonded ‘antenna’ ligand to enhance lanthanide ion luminescence sensitization.

## Graphical abstract

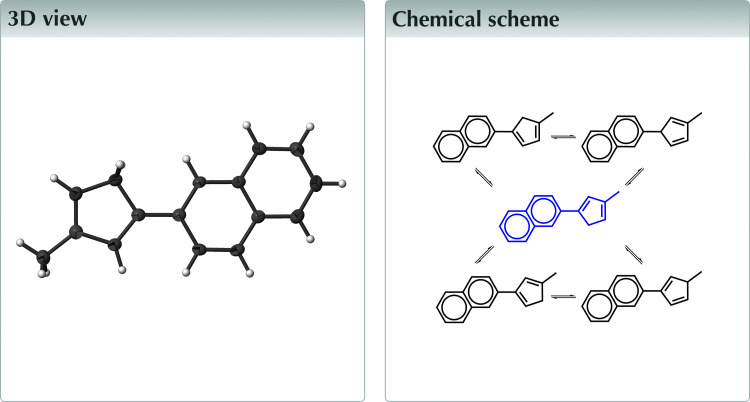

## Structure description

Aryl-substituted cyclo­penta­dienes, as functionalized cyclo­penta­dienyl ligands, complexed to rare-earth metals have been poorly explored until recently. The title compound will expand organolanthanide chemistry and is envisioned as a π-bonded ‘antenna’ ligand to enhance the photoluminescence of lanthanide coordination compounds (Roitershtein *et al.*, 2018 ▸). Similar ligands have been leveraged as effective light-harvesting π-coordinated ligands that serve as an alternative approach to traditional σ-bonded antennae for lanthanide ion luminescence sensitization (Vinogradov *et al.*, 2022 ▸).

**Figure 1:**
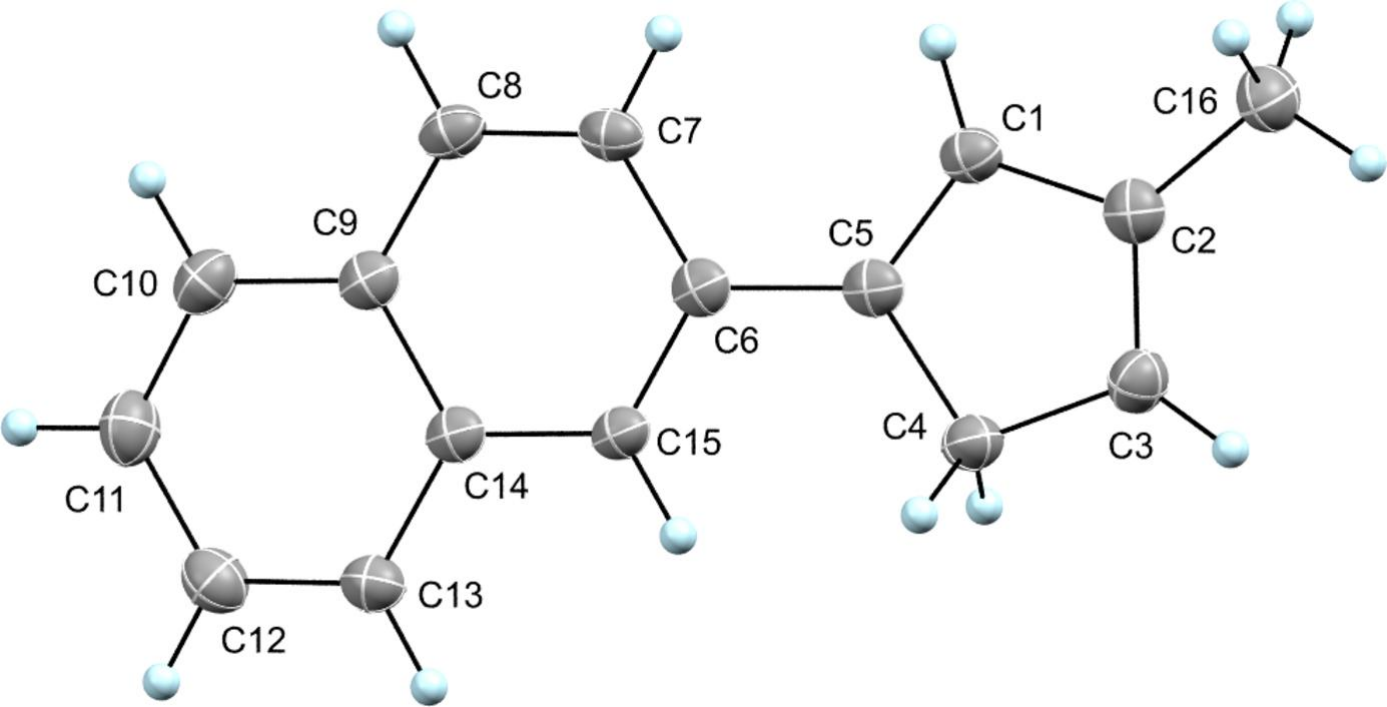
The mol­ecular structure of the title compound showing atom labeling. Displacement ellipsoids are drawn at the 50% probability level.

**Figure 2:**
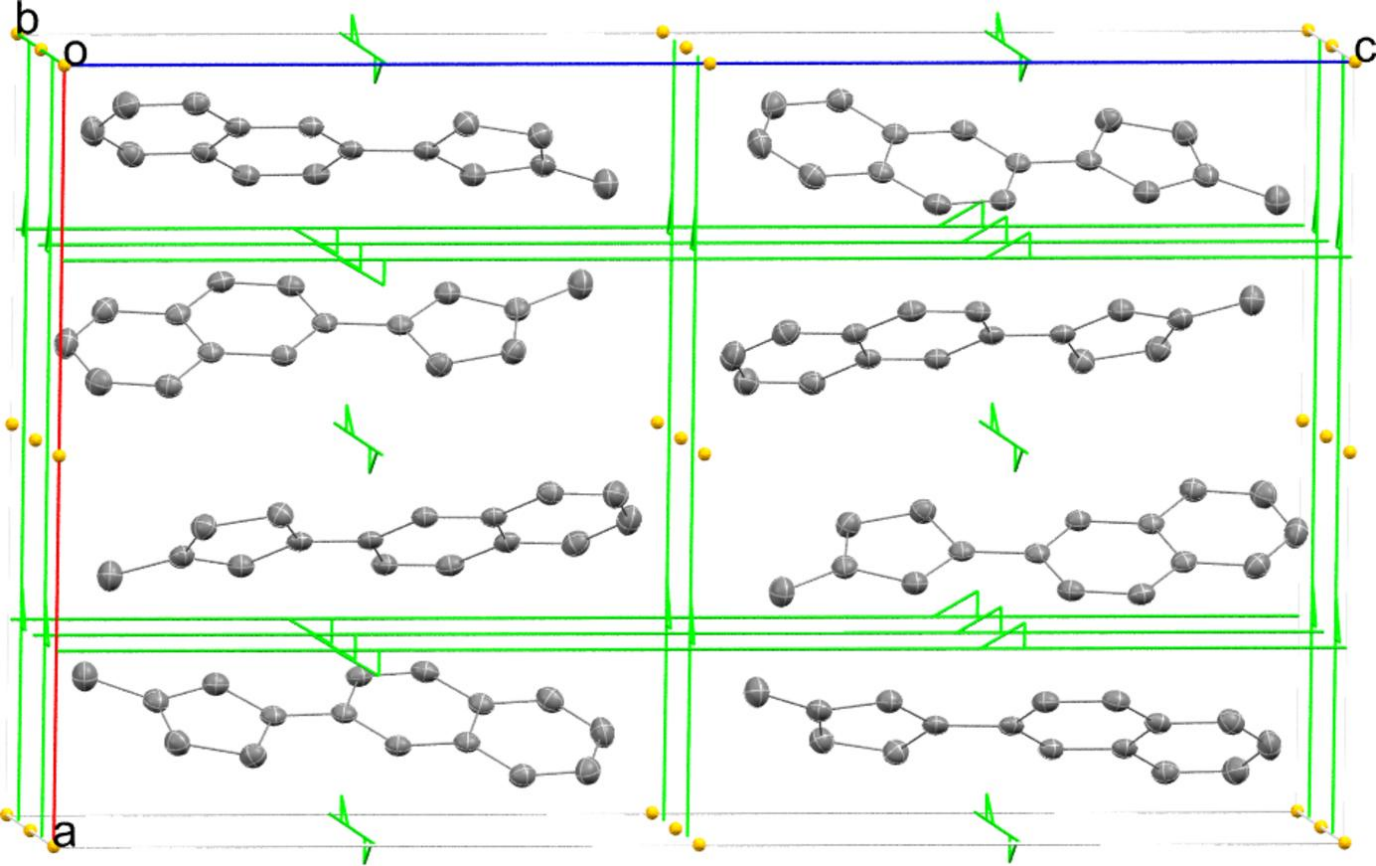
A crystal packing of the title compound. Hydrogen atoms are omitted to show symmetry elements.

The title compound was synthesized from the reaction between 2-lithium-naphthalene (made from 2-bromo-naphthalene) and 3-methyl-2-cyclo­penten-1-one following syntheses similar to Rausch (Rausch *et al.*, 2002 ▸) and Butts (Butts, 2002 ▸). The first step in this synthetic approach required very aggressive *tert*-butyl lithium to accomplish metal–halogen exchange to generate 2-lithium-naphthalene from 2-bromo-naphthalene. Since 3-methyl-2-cyclo­penten-1-one has an enolizable proton, the naphthyl-lithium generated an unreactive enolate and naphthalene as side products, which necessitated recrystal­lization of the title compound to obtain pure material. The asymmetrically disubstituted product is thermally unstable with respect to dimerization, therefore product purification must be performed quickly at room temperature with recrystallization at −30°C. Five isomers are possible with mild heating through sigmatropic rearrangement (Δ*G* ^‡^ = 26 kcal mol^−1^) (Bachrach, 1993 ▸), with one isomeric form isolated and studied by X-ray diffraction. In the crystal structure (Fig. 1 ▸), it is evident from bond distances that the title compound is a 1,3-disubstituted cyclo­penta­diene, with the methyl­ene C-atom in the 5-position (C4) and naphthyl and methyl substituents in the 1- and 3-positions, respectively. The bond distances between C1—C5 and C2—C3 are 1.364 (2) and 1.370 (3) Å, respectively, while bond distances between C1—C2, C3—C4, and C4—C5 are 1.452 (2), 1.498 (2), and 1.494 (2), respectively. There is no indication of the presence of any of the other isomers in the crystal analyzed. Fig. 2 ▸ shows a crystal packing diagram of the title compound with a canted view down along the *b* axis of the unit cell (*Z* = 8). Symmetry elements are included in the figure, with inversions (orange dots) and orthogonal screw axes (green lines with arrows). An inter­molecular C—H⋯π inter­action is also of note that seems to facilitate the observed packing, specifically between the proton of C13 from one mol­ecule and the C2—C3 bond of another mol­ecule (2.877 (3) Å).

## Synthesis and crystallization

2-Bromo­naphthalene (1.114 g, 5.379 mmol) was added to a 100 ml three-necked round-bottom flask containing a stir bar that was fitted with a gas inlet adapter, a 50 ml addition funnel, and a rubber septum; the apparatus was assembled in a glovebox under nitro­gen. Dry tetra­hydro­furan (THF, 15 ml) was added to dissolve the 2-bromo­naphthalene and *tert*-butyl­lithium (7.394 ml of a 1.7 *M* solution in pentane, 12.57 mmol, 2.3 equiv) was added to the addition funnel. The apparatus was then carefully brought out of the box and the pale-yellow solution of 2-bromo­naphthalene was cooled to −78°C in a dry ice–acetone bath with stirring under nitro­gen provided by a Schlenk-line. *tert*-Butyl­lithium was added dropwise by the addition funnel to the THF solution with stirring at −78°C. After 15 min at −78°C, the reaction was placed in an ice bath and stirred for 1 h. Then, 3-methyl-2-cyclo­penten-1-one (dried over 4 Å sieves activated by heating to 100°C for 48 h at 100 mT, 0.53 ml, 5.35 mmol) was added dropwise by syringe through the remaining rubber-stoppered neck of the three-necked round-bottom flask. The mixture was stirred for 1.5 h after which point an aqueous solution of NH_4_Cl (5 *M*, 2.4 ml, 12 mmol) was added dropwise and slowly by syringe. The reaction mixture was stirred for an additional 45 min while cooled in ice after which the volume was reduced under vacuum to ∼4 ml. The resulting semi-solid material was extracted with diethyl ether and using a separatory funnel, washed with distilled water, once with aqueous NaHCO_3_, and again with water. The organic layer was dried over MgSO_4_ and then reduced under vacuum to a viscous oil. This material was immediately stored at −30°C to prevent dimerization. To crystallize the title compound, a concentrated 50:50 diethyl ether:hexane solution of the compound was allowed to sit at −30°C overnight. Yield 75% (4.01 mmol, 0.83 g). A translucent colorless block-shaped crystal with dimensions 0.12 × 0.07 × 0.05 mm^3^ was chosen and mounted using a nylon loop for data collection. ^1^H NMR in C_6_D_6_: δ 7.77–7.58 and 7.31–7.22 (7*H*, *m*), 6.69 (1*H*, *s*), 5.91 (1*H*, *s*), 3.21 (2*H*, *s*), 1.94 (3*H*, *s*).

## Refinement

The crystal data, data collection and structure refinement details are summarized in Table 1 ▸. A number of reflections were omitted from a similar region of reciprocal space due to grazing of the incident beam by the tip of the steel shaft of the mounting pin. Beam graze was apparent from inspection of frame data.

## Supplementary Material

Crystal structure: contains datablock(s) I. DOI: 10.1107/S2414314623008568/zl4062sup1.cif

Structure factors: contains datablock(s) I. DOI: 10.1107/S2414314623008568/zl4062Isup2.hkl

Supporting information file. DOI: 10.1107/S2414314623008568/zl4062Isup3.cml

CCDC reference: 2298047

Additional supporting information:  crystallographic information; 3D view; checkCIF report

## Figures and Tables

**Table 1:** Experimental details

Crystal data	
Chemical formula	C_16_H_14_
*M*_r_	206.27
Crystal system, space group	Orthorhombic, *P* *b* *c* *a*
Temperature (K)	108
*a*, *b*, *c* (Å)	15.1769 (4), 5.8576 (2), 25.2717 (7)
*V* (Å^3^)	2246.66 (12)
*Z*	8
Radiation type	Cu *K*α
μ (mm^−1^)	0.52
Crystal size (mm)	0.12 × 0.07 × 0.05

Data collection	
Diffractometer	XtaLAB Synergy, Dualflex, HyPix
Absorption correction	Gaussian (*CrysAlis PRO*; Rigaku OD, 2023 ▸)
*T*_min_, *T*_max_	0.892, 1.000
No. of measured, independent and observed [*I* > 2σ(*I*)] reflections	8233, 2043, 1781
*R*_int_	0.052
(sin θ/λ)_max_ (Å^−1^)	0.603

Refinement	
*R*[*F* ^2^ > 2σ(*F* ^2^)], *wR*(*F* ^2^), *S*	0.054, 0.141, 1.06
No. of reflections	2043
No. of parameters	146
H-atom treatment	H-atom parameters constrained
Δρ_max_, Δρ_min_ (e Å^−3^)	0.24, −0.21

Computer programs: *CrysAlis PRO* (Rigaku OD, 2023 ▸), *SHELXT2014/5* (Sheldrick, 2015*a* ▸), *SHELXL2016/6* (Sheldrick, 2015*b* ▸), and *OLEX2* (Dolomanov *et al.*, 2009 ▸).

## full crystallographic data

### Crystal data

**Table:**

C_16_H_14_	*D*_x_ = 1.220 Mg m^−^^3^
*M**_r_* = 206.27	Cu *K*α radiation, λ = 1.54184 Å
Orthorhombic, *P**b**c**a*	Cell parameters from 4778 reflections
*a* = 15.1769 (4) Å	θ = 3.5–76.0°
*b* = 5.8576 (2) Å	µ = 0.52 mm^−^^1^
*c* = 25.2717 (7) Å	*T* = 108 K
*V* = 2246.66 (12) Å^3^	Block, clear light colourless
*Z* = 8	0.12 × 0.07 × 0.05 mm
*F*(000) = 880	

### Data collection

**Table:**

XtaLAB Synergy, Dualflex, HyPix diffractometer	2043 independent reflections
Radiation source: micro-focus sealed X-ray tube, PhotonJet (Cu) X-ray Source	1781 reflections with *I* > 2σ(*I*)
Mirror monochromator	*R*_int_ = 0.052
Detector resolution: 10.0000 pixels mm^-1^	θ_max_ = 68.5°, θ_min_ = 3.5°
ω scans	*h* = −18→17
Absorption correction: gaussian (CrysAlisPro; Rigaku OD, 2023)	*k* = −7→5
*T*_min_ = 0.892, *T*_max_ = 1.000	*l* = −30→18
8233 measured reflections	

### Refinement

**Table:**

Refinement on *F*^2^	Primary atom site location: dual
Least-squares matrix: full	Hydrogen site location: inferred from neighbouring sites
*R*[*F*^2^ > 2σ(*F*^2^)] = 0.054	H-atom parameters constrained
*wR*(*F*^2^) = 0.141	*w* = 1/[σ^2^(*F*_o_^2^) + (0.067*P*)^2^ + 1.5617*P*] where *P* = (*F*_o_^2^ + 2*F*_c_^2^)/3
*S* = 1.06	(Δ/σ)_max_ = 0.001
2043 reflections	Δρ_max_ = 0.24 e Å^−^^3^
146 parameters	Δρ_min_ = −0.21 e Å^−^^3^
0 restraints	

### Special details

**Table:**

Geometry. All esds (except the esd in the dihedral angle between two l.s. planes) are estimated using the full covariance matrix. The cell esds are taken into account individually in the estimation of esds in distances, angles and torsion angles; correlations between esds in cell parameters are only used when they are defined by crystal symmetry. An approximate (isotropic) treatment of cell esds is used for estimating esds involving l.s. planes.
Refinement. All non-hydrogen atoms were refined anisotropically and all H atom positions were calculated geometrically and refined using a riding model.

### Fractional atomic coordinates and isotropic or equivalent isotropic displacement parameters (Å^2^)

**Table:**

	*x*	*y*	*z*	*U*_iso_*/*U*_eq_	
C1	0.33584 (11)	0.4499 (3)	0.83770 (7)	0.0231 (4)	
H1	0.304514	0.310583	0.833690	0.028*	
C2	0.35225 (11)	0.5625 (3)	0.88793 (7)	0.0246 (4)	
C3	0.39827 (12)	0.7593 (3)	0.87825 (7)	0.0266 (4)	
H3	0.416965	0.865805	0.904289	0.032*	
C4	0.41436 (11)	0.7795 (3)	0.81995 (7)	0.0250 (4)	
H4A	0.387308	0.920341	0.805653	0.030*	
H4B	0.478272	0.781264	0.812124	0.030*	
C5	0.37161 (10)	0.5719 (3)	0.79708 (7)	0.0212 (4)	
C6	0.37066 (10)	0.5194 (3)	0.74040 (6)	0.0206 (4)	
C7	0.33159 (10)	0.3124 (3)	0.72152 (7)	0.0221 (4)	
H7	0.307475	0.207164	0.746172	0.027*	
C8	0.32835 (11)	0.2636 (3)	0.66883 (7)	0.0233 (4)	
H8	0.301906	0.124974	0.657483	0.028*	
C9	0.36362 (10)	0.4153 (3)	0.63058 (7)	0.0219 (4)	
C10	0.36012 (12)	0.3717 (3)	0.57556 (7)	0.0274 (4)	
H10	0.332560	0.236549	0.563016	0.033*	
C11	0.39603 (13)	0.5223 (3)	0.54022 (7)	0.0311 (4)	
H11	0.393374	0.490849	0.503378	0.037*	
C12	0.43706 (12)	0.7242 (3)	0.55830 (7)	0.0292 (4)	
H12	0.462141	0.827380	0.533505	0.035*	
C13	0.44094 (11)	0.7723 (3)	0.61096 (7)	0.0245 (4)	
H13	0.468654	0.908877	0.622521	0.029*	
C14	0.40416 (10)	0.6212 (3)	0.64870 (7)	0.0207 (4)	
C15	0.40649 (10)	0.6670 (3)	0.70371 (6)	0.0207 (4)	
H15	0.433592	0.803662	0.715715	0.025*	
C16	0.32435 (13)	0.4703 (3)	0.93989 (7)	0.0311 (5)	
H16A	0.260298	0.449195	0.940060	0.047*	
H16B	0.340994	0.577529	0.967929	0.047*	
H16C	0.353315	0.323142	0.946051	0.047*	

### Atomic displacement parameters (Å^2^)

**Table:**

	*U*^11^	*U*^22^	*U*^33^	*U*^12^	*U*^13^	*U*^23^
C1	0.0203 (8)	0.0213 (9)	0.0278 (9)	0.0000 (7)	−0.0002 (6)	−0.0007 (7)
C2	0.0210 (8)	0.0266 (9)	0.0261 (9)	0.0042 (7)	−0.0015 (6)	0.0005 (7)
C3	0.0263 (9)	0.0281 (9)	0.0256 (9)	−0.0008 (7)	−0.0020 (7)	−0.0027 (8)
C4	0.0245 (9)	0.0230 (9)	0.0275 (9)	−0.0030 (7)	0.0004 (7)	−0.0011 (7)
C5	0.0152 (8)	0.0210 (9)	0.0274 (9)	0.0034 (6)	−0.0005 (6)	0.0007 (7)
C6	0.0146 (8)	0.0211 (8)	0.0263 (9)	0.0041 (6)	−0.0005 (6)	−0.0007 (7)
C7	0.0172 (8)	0.0205 (9)	0.0288 (9)	0.0005 (6)	0.0010 (6)	0.0029 (7)
C8	0.0186 (8)	0.0199 (9)	0.0315 (9)	−0.0003 (7)	−0.0025 (7)	−0.0017 (7)
C9	0.0179 (8)	0.0205 (9)	0.0273 (9)	0.0038 (7)	−0.0021 (6)	−0.0004 (7)
C10	0.0269 (9)	0.0257 (9)	0.0298 (9)	0.0013 (7)	−0.0047 (7)	−0.0039 (8)
C11	0.0364 (10)	0.0344 (10)	0.0226 (8)	0.0041 (8)	−0.0034 (7)	−0.0013 (8)
C12	0.0298 (10)	0.0303 (10)	0.0274 (9)	0.0016 (8)	0.0010 (7)	0.0061 (8)
C13	0.0218 (8)	0.0224 (9)	0.0292 (9)	0.0004 (7)	0.0007 (7)	0.0023 (7)
C14	0.0159 (8)	0.0187 (8)	0.0274 (9)	0.0034 (6)	−0.0005 (6)	−0.0005 (7)
C15	0.0171 (8)	0.0179 (8)	0.0269 (8)	0.0004 (6)	−0.0005 (6)	−0.0018 (7)
C16	0.0362 (10)	0.0320 (10)	0.0251 (9)	−0.0010 (8)	−0.0004 (7)	0.0013 (8)

### Geometric parameters (Å, º)

**Table:**

C1—H1	0.9500	C8—C9	1.418 (2)
C1—C2	1.452 (2)	C9—C10	1.415 (2)
C1—C5	1.364 (2)	C9—C14	1.429 (2)
C2—C3	1.370 (3)	C10—H10	0.9500
C2—C16	1.482 (2)	C10—C11	1.368 (3)
C3—H3	0.9500	C11—H11	0.9500
C3—C4	1.498 (2)	C11—C12	1.413 (3)
C4—H4A	0.9900	C12—H12	0.9500
C4—H4B	0.9900	C12—C13	1.362 (2)
C4—C5	1.494 (2)	C13—H13	0.9500
C5—C6	1.465 (2)	C13—C14	1.416 (2)
C6—C7	1.432 (2)	C14—C15	1.416 (2)
C6—C15	1.379 (2)	C15—H15	0.9500
C7—H7	0.9500	C16—H16A	0.9800
C7—C8	1.363 (2)	C16—H16B	0.9800
C8—H8	0.9500	C16—H16C	0.9800
			
C2—C1—H1	124.7	C8—C9—C14	118.23 (15)
C5—C1—H1	124.7	C10—C9—C8	122.86 (16)
C5—C1—C2	110.60 (16)	C10—C9—C14	118.91 (16)
C1—C2—C16	124.06 (17)	C9—C10—H10	119.7
C3—C2—C1	108.27 (15)	C11—C10—C9	120.68 (17)
C3—C2—C16	127.66 (17)	C11—C10—H10	119.7
C2—C3—H3	125.5	C10—C11—H11	119.9
C2—C3—C4	108.97 (15)	C10—C11—C12	120.28 (17)
C4—C3—H3	125.5	C12—C11—H11	119.9
C3—C4—H4A	110.9	C11—C12—H12	119.7
C3—C4—H4B	110.9	C13—C12—C11	120.54 (17)
H4A—C4—H4B	108.9	C13—C12—H12	119.7
C5—C4—C3	104.22 (14)	C12—C13—H13	119.6
C5—C4—H4A	110.9	C12—C13—C14	120.81 (17)
C5—C4—H4B	110.9	C14—C13—H13	119.6
C1—C5—C4	107.94 (15)	C13—C14—C9	118.78 (15)
C1—C5—C6	128.47 (16)	C13—C14—C15	122.21 (16)
C6—C5—C4	123.59 (15)	C15—C14—C9	119.01 (15)
C7—C6—C5	120.52 (15)	C6—C15—C14	122.11 (16)
C15—C6—C5	121.47 (15)	C6—C15—H15	118.9
C15—C6—C7	118.01 (15)	C14—C15—H15	118.9
C6—C7—H7	119.4	C2—C16—H16A	109.5
C8—C7—C6	121.24 (16)	C2—C16—H16B	109.5
C8—C7—H7	119.4	C2—C16—H16C	109.5
C7—C8—H8	119.3	H16A—C16—H16B	109.5
C7—C8—C9	121.38 (16)	H16A—C16—H16C	109.5
C9—C8—H8	119.3	H16B—C16—H16C	109.5
